# NKG2D Engagement Alone Is Sufficient to Activate Cytokine-Induced Killer Cells While 2B4 Only Provides Limited Coactivation

**DOI:** 10.3389/fimmu.2021.731767

**Published:** 2021-10-07

**Authors:** Xiaolong Wu, Amit Sharma, Johannes Oldenburg, Hans Weiher, Markus Essler, Dirk Skowasch, Ingo G. H. Schmidt-Wolf

**Affiliations:** ^1^ Department of Integrated Oncology, Center of Integrated Oncology (CIO) Bonn, University Hospital Bonn, Bonn, Germany; ^2^ Department of Neurosurgery, University Hospital Bonn, Bonn, Germany; ^3^ Institute of Experimental Hematology and Transfusion Medicine, University Hospital Bonn, Bonn, Germany; ^4^ Department of Applied Natural Sciences, Bonn-Rhein-Sieg University of Applied Sciences, Rheinbach, Germany; ^5^ Department of Nuclear Medicine, University Hospital Bonn, Bonn, Germany; ^6^ Department of Internal Medicine II, University Hospital Bonn, Bonn, Germany

**Keywords:** cytokine-induced killer (CIK) cells, NKG2D, MICA/B, 2B4, LFA-1

## Abstract

Cytokine-induced killer (CIK) cells are an *ex vivo* expanded heterogeneous cell population with an enriched NK-T phenotype (CD3+CD56+). Due to the convenient and relatively inexpensive expansion capability, together with low incidence of graft *versus* host disease (GVHD) in allogeneic cancer patients, CIK cells are a promising candidate for immunotherapy. It is well known that natural killer group 2D (NKG2D) plays an important role in CIK cell-mediated antitumor activity; however, it remains unclear whether its engagement alone is sufficient or if it requires additional co-stimulatory signals to activate the CIK cells. Likewise, the role of 2B4 has not yet been identified in CIK cells. Herein, we investigated the individual and cumulative contribution of NKG2D and 2B4 in the activation of CIK cells. Our analysis suggests that (a) NKG2D (not 2B4) is implicated in CIK cell (especially CD3+CD56+ subset)-mediated cytotoxicity, IFN-γ secretion, E/T conjugate formation, and degranulation; (b) NKG2D alone is adequate enough to induce degranulation, IFN-γ secretion, and LFA-1 activation in CIK cells, while 2B4 only provides limited synergy with NKG2D (e.g., in LFA-1 activation); and (c) NKG2D was unable to costimulate CD3. Collectively, we conclude that NKG2D engagement alone suffices to activate CIK cells, thereby strengthening the idea that targeting the NKG2D axis is a promising approach to improve CIK cell therapy for cancer patients. Furthermore, CIK cells exhibit similarities to classical invariant natural killer (iNKT) cells with deficiencies in 2B4 stimulation and in the costimulation of CD3 with NKG2D. In addition, based on the current data, the divergence in receptor function between CIK cells and NK (or T) cells can be assumed, pointing to the possibility that molecular modifications (e.g., using chimeric antigen receptor technology) on CIK cells may need to be customized and optimized to maximize their functional potential.

## Introduction

Cytokine-induced killer (CIK) cells, as *ex vivo* expanded lymphocytes generated from peripheral blood mononuclear cells (PBMCs) in the presence of a cocktail of stimuli (IFN-γ, anti-CD3 antibody, IL-2, IL-1β), were first introduced by Ingo Schmidt-Wolf and colleagues in 1991 (1). After 14–21 days of expansion, CIK cells become a heterogeneous population of lymphocytes composed of a majority of CD3+CD56− T cells and CD3+CD56+ cells and a minor fraction of CD3−CD56+ natural killer (NK) cells (2). Under this culture condition, the CD3+CD56+ subset is primarily derived from CD3+CD56− T cells and can be enriched in large numbers with great cytotoxicity (3). In comparison to lymphocyte-activated killer (LAK) cells, CIK cells exhibit a higher proliferation rate and possess superior *in vivo* antitumor activity (3). Though CIK cells resemble NK-like T cells, in contrast to classical invariant natural killer T (iNKT) cells, they consist of a high proportion of CD8 cells with a heterogeneous Vβ repertoire (4). With phenotypic and functional similarities to cytotoxic T cells and NK cells, CIK cells are endowed with potent cytotoxicity against a broad range of tumors (solid and hematologic origins) in both MHC-restricted and MHC-unrestricted manners (5–7). CIK cell therapy has shown some encouraging results in clinical trials for advanced cancer patients with favorably low incidence of adverse effects, even when infused as an allogeneic product (8–12). In a recent study, Merker et al. reported that CIK cell therapy induced a higher complete remission (CR) rate in patients with relapsing hematological malignancies after allogeneic HSCTs than donor-derived lymphocyte infusion (DLI) (53% and 29%, respectively), while relapse occurred in 47% and 71%, respectively (13). More importantly, no concurrent salvage therapies were used in this study, which could probably better interpret the efficacy of CIK cell therapy.

It is well established that the cytotoxic lymphocytes kill the target cells by releasing the contents of the secretory lysosomes at the immune synapse (a process called degranulation). Besides, the interaction of LFA-1 (lymphocyte function-associated antigen) on effector cells with its ligands (intracellular adhesion molecules [ICAMs]) on target cells contributes to stable adhesion, immunological synapse formation, polarization of lytic granules, and subsequent killing of the target cell. In agreement with this, we previously showed that stimulation by the CIK recognition structure in concert with LFA-1 could lead to granule-dependent cytolysis (14). Subsequently, some activating NK receptors were detected as recognition structures of CIK cells, including natural killer group 2D (NKG2D), DNAX accessory molecule-1 (DNAM-1), NKp30, and CD16 (7, 15–17). Notably, NKG2D is considered among them to be the main contributor to the MHC-unrestricted cytolysis of CIK cells against various types of tumors (15, 18, 19), probably through the disulfide adaptor protein 10 (DAP-10)-mediated signaling (15, 20). Like other effector cells (e.g., cytotoxic T cells and NK cells), CIK cells kill targets after activation most likely by direct granule-dependent cytolysis, as expanded CIK cells from perforin KO mice did not exhibit cytolytic activity against tumor cell lines (21). In addition, the cytotoxic activity of CIK cells is also found to be mediated by the program cell death system Fas-FasL and tumor necrosis factor-related apoptosis-inducing ligand (TRAIL) (22, 23).

NKG2D is primarily a C-type lectin-like receptor capable of activating NK cells and co-stimulating CD8+ T cells (24, 25). In humans, the NKG2D receptor recognizes a number of ligands (MICA/B, ULBP1-6) that are usually absent (or present in low amounts) on the surface of normal cells but are induced or upregulated by various stress signals (e.g., infection, transformation, DNA damage) (26), thus making them more susceptible to the immune system. It is well established that blocking the NKG2D receptor on CIK cells can partially reduce cytolytic activity against NKG2D-bearing tumor cells (7, 15, 18, 19). Therefore, it would be of interest to know if NKG2D ligation alone can lead to the activation of CIK cells, since the interaction of NKG2D, 2B4, and LFA-1 has been defined as a minimum requirement for the induction of natural cytotoxicity (27). As with NKG2D, 2B4 (CD244) is another well-characterized NK cell activation receptor that belongs to the lymphocyte signaling activation molecule (SLAM) family (28). To our knowledge, the peculiar role of 2B4 in CIK cells remains unknown.

Considering this, herein, we aim to investigate (1) whether engagement of NKG2D alone is sufficient to trigger and activate CIK cells and (2) what role 2B4 alone and in combination with NKG2D may play in the activation of CIK cells. To achieve this, we used multiple cell lines and diverse methodological approaches (cytotoxicity assay, conjugate assay, CD107a degranulation assay, ligand complex-based adhesion assay, and ELISA).

## Materials and Methods

### Cell Culture and Antibodies

CIK cells were generated, as described previously (1). Briefly, PBMCs were isolated from healthy donors registered at the blood bank of University Hospital Bonn by gradient density centrifugation using Pancoll (Pan-Biotech, Aidenbach, Bavaria, Germany). PBMCs were first seeded at 3 × 10^6^ cells/ml in a 75 cm^2^ flask for 2 h to remove the monocytes. Subsequently, on day 0, 1000 U/ml IFN-γ was added, followed by the addition of 50 ng/ml anti-CD3, 600 U/ml IL-2, and 100 U/ml IL-1β on day 1. Cells were incubated at 37°C, 5% CO_2_, and humidified atmosphere and subcultured every 3 days with fresh medium supplemented with 600U IL-2 at 0.5–1 × 10^6^ cells/ml. After 2 weeks of *ex vivo* expansion, CIK cells were collected for the experiments.

RPMI-1640 (Pan-Biotech, Aidenbach, Bavaria, Germany) medium supplemented with 10% heat-inactivated FBS (Sigma-Aldrich Chemie GmbH, Munich, Germany) and 1% penicillin/streptomycin (P/S) (Gibco, Schwerte, Germany) was used for the cell cultures. Four cell lines: K562 (leukemia), Raji (lymphoma), SU-DHL-4 (lymphoma) (all from DSMZ, Braunschweig, Germany), and mouse mastocytoma P815 (ATCC) were primarily used in this study. Of note, as discussed previously, the heterogeneity between cancer cell lines (in addition to genetic–epigenetic variations) and inter-individual differences can lead to experimental discrepancies (29); in our study, with four stable cell lines and four donors, no inconsistencies were observed. Concerning antibodies (Abs) and cytokines, anti-CD3 (OKT3) and anti-2B4 (C1.7) were purchased from eBioscience (Inc. San Diego, CA, U.S.A.). Anti-IgG1 (MOPC-21), anti-NKG2D (1D11), anti-CD3-FITC (OKT3), anti-CD3-APC (OKT3), anti-CD3-PerCP-Cy5.5 (OKT3), anti-CD56-PE (5.1H11), anti-CD56-FITC (5.1H11), anti-MICA/B-APC (6D4), anti-NKG2D-APC (1D11), anti-2B4-PE (C1.7), anti-LFA-1-APC (m24), and their respective isotypes were purchased from Biolegend (Koblenz, Germany). Additionally, anti-CD107a-APC (H4A3), anti-CD48-PE (BJ40), and the respective isotypes were purchased from BD Bioscience (Heidelberg, Germany). All cytokines (IFN-γ, IL-2, and IL-1β) were purchased from ImmunoTools (GmbH, Aidenbach, Bavaria, Germany).

### Conjugate Assay

We labeled target cells with CFSE (Thermo Fisher Scientific, Eugene, U.S.A.) and effector cells with CellTrace Violet (Thermo Fisher Scientific, Eugene, U.S.A.) in 1 ml of PBS (5 min, 37°C in the dark), followed by washing three times with 5 ml of culture medium with 10% FBS. CFSE-labeled P815 cells were incubated with the indicated Abs (IgG1, anti-NKG2D, and anti-2B4) using a concentration of 5 µg/ml (30 min) for the redirected conjugate assay. Likewise, purple-labeled CIK cells were incubated with the indicated Abs (IgG1, anti-NKG2D, and anti-2B4) using a concentration of 10 µg/ml (30 min) for the blocking experiments. Subsequently, 2.5 × 10^4^ CFSE-labeled target cells were co-cultured with Violet-labeled CIK cells at an E/T ratio of 5:1 specifically in 1.5-ml Eppendorf tubes in the presence of 7AAD (final concentration 0.5 µg/ml, Biolegend, Koblenz, Germany). Cells were gently mixed and centrifuged (200 g, 2 min) to facilitate cell-to-cell contact. After brief incubation (10 min, 37°C water bath), cells were vortexed (5 s) to break nonspecific binding, fixed with 1% paraformaldehyde (5 min), and then measured with fluorescence-activated cell sorting (FACS) Canto II (BD Biosciences, Heidelberg, Germany).

### Cytotoxicity Assay

Flow cytometry-based cytotoxicity was performed, as described previously (30). Briefly, the target cells were labeled with CFSE (1 ml PBS, 5 min, 37°C in the dark), followed by three washings with culture medium. CFSE-labeled P815 cells were incubated at a concentration of 5 µg/ml (except anti-CD3, 0.05 µg/ml) for 30 min with the indicated Abs (IgG1, anti-NKG2D, anti-2B4, and anti-CD3) to perform the redirected cytolysis assay. For blocking experiments, CIK cells were pre-incubated with 10 µg/ml anti-NKG2D Ab or anti-2B4 Ab (30 min). Subsequently, 2 × 10^4^ target cells were co-cultured with CIK cells at varied E/T ratios, following 6–8 h of culturing, and the cells were stained with a viability dye Hoechst 33258 (Cayman Chemical, Hamburg, Germany) and quantified using FACS.

### CD107a Degranulation Assay

The standard degranulation assay was performed using a lysosomal marker CD107a.

For cell-mediated stimulation, P815 cells were pre-incubated with the indicated Abs (IgG1, anti-NKG2D, anti-2B4; 5 µg/ml for 30 min) and then co-cultured with CIK cells at an E/T ratio of 5:1 in the presence of anti-CD107a (1:100) and GolgiStop (1:1500, BD Biosciences, Heidelberg, Germany) in 96-well U-bottom plates. Next, the cells were centrifuged (100 g, 2 min) and incubated (5 h, 37°C, 5% CO_2_).

For plate-bound antibody stimulation, Abs were coated at concentration of 5 µg/ml for 3 h (37°C) on the high-binding 96-well flat-bottom plate (Corning, Berlin, Germany). After incubation, PBS was aspirated and CIK cells (2 × 10^5^/well) were added with the culture medium (37°C, 5% CO_2_). Next, anti-CD107a-APC (1:100) and GolgiStop (1:1500) were added after 1 h and further incubation for 4 h was considered.

Subsequently, the cells were washed twice with PBS and stained with anti-CD3-FITC and anti-CD56-PE. At the end, the percentage of CD107a-positive cells in bulk CIK cells or in subsets (CD3+CD56− and CD3+CD56+) was measured by FACS.

### Ligand Complex-Based Adhesion Assay

A ligand complex-based adhesion assay (LC-AA) was performed, as described previously (31). A base buffer containing PBS (0.5% BSA) with or without cations (1mM CaCl_2_ and 2mM MgCl_2_) was prepared for all the incubation steps. Also, 50 µg/ml recombinant human ICAM-1–Fc chimera (R&D Systems, Minneapolis, USA) and F(ab)2 fragments of goat anti-human Fcγ fragment (160 µg/ml FITC-labeled, Jackson Immuno-Research) were mixed (cation-free buffer, 30 min, RT) to prepare ICAM-1–Fc complexes. CD99-Fc (R&D) was used as a negative control for gating instead of ICAM-1–Fc. CIK cells were incubated (10 min) with all primary Abs at the concentration of 2 µg/ml (except anti-CD3, 0.5 µg/ml), followed by washing and resuspension of the cells in the buffer and ICAM-1–Fc complex (dilution 1:20). The cross-linking with goat anti-mouse antibody (5 µg/ml) was performed at a 37°C water bath. The stimulation controls were performed by adding PMA (10 nM, Sigma), and the cells were fixed after 10 min by addition of paraformaldehyde (final concentration 1%). In some cases, cells were stained with CD3 and CD56 markers to identify the CIK subsets after stimulation. Afterwards, the cells were stained with Hoechst 33258 and measured by FACS.

In order to confirm whether LC-AA assay is able to detect both affinity and avidity changes of LFA-1 in CIK cells, a specific clone m24 antibody with the ability to detect the LFA-1 in high-affinity conformation was used. CIK cells were stimulated with PMA (10 nM) or Mg2+ (10 nM) for 10 min either in the presence of the ICAM-1–Fc complex (dilution 1:20) or m24-APC (dilution 1:100). After stimulation, cells were stained with Hoechst 33258 and measured by FACS.

### Function Inhibition by Using Signaling Pathway Inhibitors

Where indicated, CIK cells were preincubated with inhibitors separately (30 µM PP1, 100 nM wortmannin, and 2.5 µM U73122, source: Biomol) for 30 min (37°C), and these inhibitors were retained in the medium during the stimulation or culture process.

### Cell Sorting

The CIK cells were washed twice with PBS buffer containing BSA (0.5%) and EDTA (2 mM), incubated with CD56-conjugated microbeads (Miltenyi Biotec, Auburn, CA), and passed through a LS+ magnetic-activated cell sorting (MACS) separation column, according to the manufacturer’s instructions (Miltenyi). The eluant contained the CD3+ CD56− subset, while the positively selected CD56+ cells were collected by flushing the column. Because CIK cells usually contain a small proportion of CD3−CD56+ NK cells, which varies depending on the donor, CIK cells with a low proportion of NK cells were selected to ensure that the purity of CD3+CD56+ cells after sorting was greater than 95% to avoid the influence of NK cells.

### ELISA

CIK cells (1 × 10^6^/well) were stimulated by plate-bound Abs (precoated at 5 µg/ml, 37°C, 3 h) in the culture medium for 24 h at 37°C, 5% CO_2_. Thereafter, the cell-free supernatant was collected to perform sandwich ELISA assay (IFN-γ kit, Invitrogen, Camarillo, CA, USA), according to the manufacturer’s instructions.

### Statistical Analysis

All experiments were conducted in triplicate and repeated at least three times. Experiments with CIK cells were performed with at least three independent donors. FACS data sets were analyzed using FlowJo V10.6 software (FlowJo, LLC, Ashland, Oregon, U.S.A.). Statistical analyses were performed using GraphPad Prism v.8.0 (GraphPad Software, Inc., San Diego, CA, U.S.A.). The data groups were compared using one-way or two-way analysis of variance (ANOVA) with Bonferroni’s *post-hoc* test. *p*-values < 0.05 were considered significant differences and are marked: *p < 0.05; **p < 0.01; ***p < 0.001; ****p < 0.0001; ns = not significant.

## Results

### NKG2D and 2B4 Expression Levels Elevate Over Time in CIK Culture

**Figure 1:**
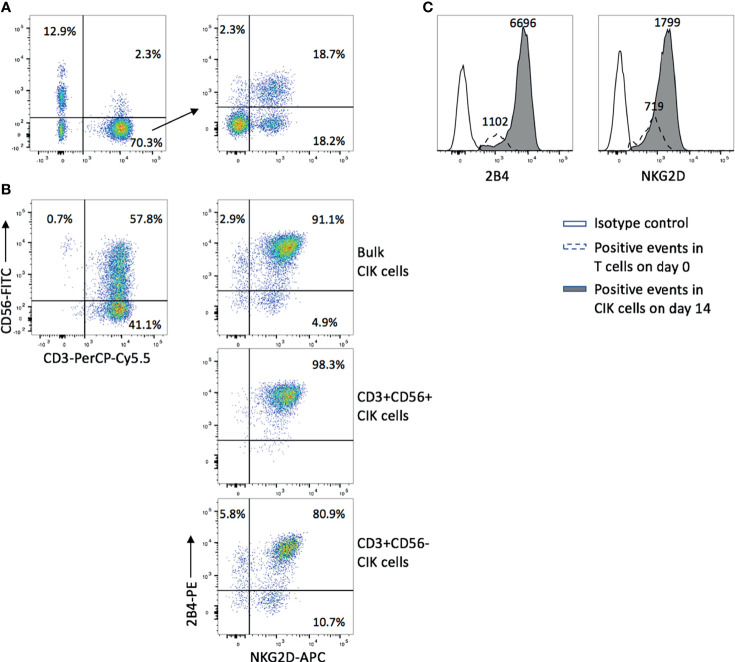
NKG2D and 2B4 expression levels elevate over time in CIK culture. CIK cells were generated by culturing fresh PBMCs in the presence of IFN-γ, anti-CD3 antibody, IL-1β, and IL-2 for 14 days. NKG2D and 2B4 expression on T cells in freshly isolated PBMCs on day 0 **(A)** and CIK cells on day 14 **(B)** were measured by flow cytometry after staining with anti-CD3-PerCP-Cy5.5, anti-CD56-FITC, anti-NKG2D-APC, and anti-2B4-PE antibodies. **(A)** Dot plots show the phenotype of freshly isolated lymphocytes (left) and the expression of NKG2D (36.9%) and 2B4 (21.0%) within the CD3+CD56− T-cell population (right). **(B)** Dot plots show the phenotype of mature CIK cells (top left), and the elevated expression of NKG2D and 2B4 on bulk CIK cells (top right), CD3+CD56+ subset (middle), and CD3+CD56− subset (bottom). **(C)** The intensity of NKG2D and 2B4 on bulk CIK cells was increased over 14-day culture in comparison to the fresh T cells. Number indicates the median fluorescence intensity. The figure shows data from one representative from four donors.

We first confirmed that all CD3−CD56+ NK cells in PBMCs expressed 2B4 while NKG2D presented on most of NK cells (**Figure S1**). As it was previously described that the majority of CD3+CD56+ cytotoxic cells in CIK cultures were derived from CD3+CD56− T cells (3), we focused mainly on CD3+CD56− T cells (day 0) and detected the expression of both NKG2D (36.9%) and 2B4 (21.0%) (**Figure 1A**). Interestingly, after 14 days of *ex vivo* expansion, not only the percentage of CD3+C56+ cells increased (57.8%), but also the expression of NKG2D (96.0%) and 2B4 (94.0%) in the bulk CIK cells (**Figure 1B**, upper right). Of note, 91.1% cells coexpressed NKG2D and 2B4. A similar expression pattern was observed in the subsets of CIK cells, with higher coexpression of NKG2D and 2B4 in CD3+CD56+ cells (98.3%, **Figure 1B**, middle) *versus* CD3+CD56− cells (80.9%, **Figure 1B**, bottom). In comparison to the precursor T cells, CIK cells gained an increase in the expression of both receptors, not only in a higher percentage but in a higher intensity (**Figure 1C**), which indicates that these two receptors are dramatically upregulated on CIK cells during the *ex vivo* expansion. The data shown are representative results from four donors.

### Blockade of NKG2D but not 2B4 Attenuates the CIK Cell-Mediated Cytotoxicity and E/T Conjugate Formation

**Figure 2:**
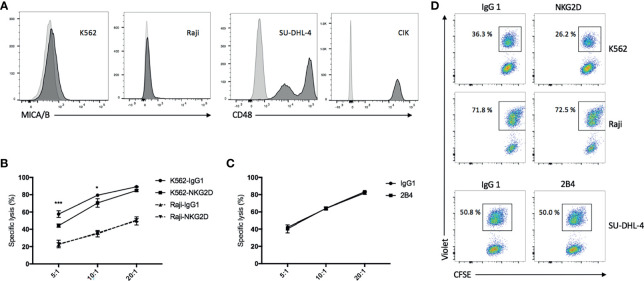
Blockade of NKG2D but not 2B4 attenuates the CIK cell-mediated cytotoxicity and E/T conjugate formation. **(A)** FACS histograms demonstrating the presence of MICA/B on K562 cells and absence on Raji cells (left) and the presence of CD48 on all SU-DHL-4 cells and CIK cells (right). **(B)** Blocking cytotoxicity was performed by preincubation of CIK cells with IgG1 isotype control or anti-NKG2D (clone 1D11) antibodies (10 µg/ml) for 30 min prior to coculture with CFSE-labeled K562 or Raji cells. After 6–8 h of coculture at indicated effector/target (E/T) ratios, cells were stained with viability dye Hoechst 33258 and measured on flow cytometry. **(C)** Cytotoxicity was performed as described in “B” by preincubation of CIK cells with IgG1 control or anti-2B4 (clone C1.7) antibodies (10 µg/ml) for 30 min prior to coculture with CFSE-labeled SU-DHL-4 cells at indicated E/T ratios. **(D)** CellTrace Violet-labeled CIK cells were preincubated with IgG1 control or anti-NKG2D or anti-2B4 antibodies (10 µg/ml) for 30 min and then cocultured with CFSE-labeled K562 (top panel) or Raji (middle panel) or SU-DHL-4 cells (bottom panel) at a 5:1 E/T ratio in the presence of 7AAD for 10 min at 37°C. As next, cells were fixed with 1% paraformaldehyde and measured on flow cytometry. CFSE+Violet+ double-positive events were considered the E/T conjugate. Plots show the percentage of E/T conjugate within the CFSE+ target gate. Aforementioned data are shown as mean ± SD of triplicates per condition and one representative of three independent experiments. *p < 0.05, ***p < 0.001 calculated by two-way ANOVA, Bonferroni’s *post-hoc* test.

We observed that MICA/B was expressed on part of K562 cells but absent on Raji cells (**Figure 2A**, left panel). Expectedly, blockade of NKG2D on CIK cells with anti-NKG2D resulted in a reduction in both cytotoxicity and E/T conjugate formation in K562 cells but not in Raji cells (**Figures 2B, D**, top and middle panels). Conversely, CD48 (the ligand of 2B4) was expressed on all SU-DHL-4 and CIK cells (**Figure 2A**, right panel), and no decline in cytotoxicity and E/T conjugation was observed when CIK cells were preincubated with anti-2B4 (**Figures 2C, D**, bottom panel), thus suggesting that NKG2D (not 2B4) is directly implicated in CIK cell-mediated cytotoxicity and E/T conjugate formation.

### Engagement of NKG2D (Not 2B4) Increases the CIK Cell-Mediated Cytotoxicity, Degranulation, IFN-γ Secretion, and E/T Binding Against P815 Cells

**Figure 3:**
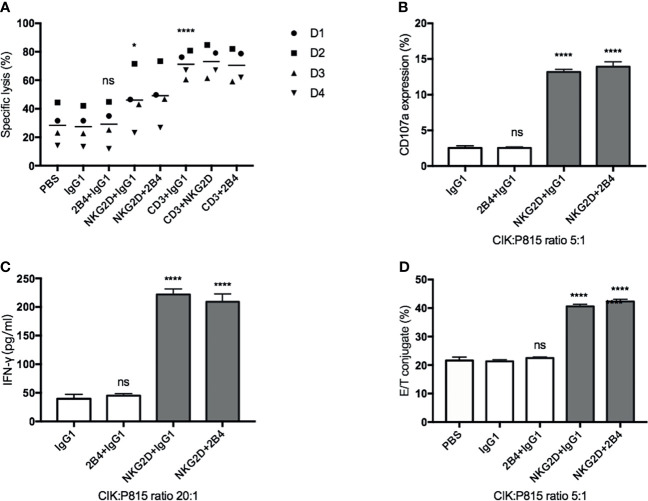
NKG2D (not 2B4) increases the CIK cell-mediated cytotoxicity, degranulation, IFN-γ secretion, and E/T binding against P815 cells. **(A)**. CFSE-labeled P815 cells were preincubated with indicated antibodies at a concentration of 5 µg/ml (except anti-CD3, 0.05 µg/ml) for 30 min before coculture with CIK cells. After 6–8 h of coculture at a 20:1 E/T ratio, cells were stained with Hoechst 33258 and measured on flow cytometry. **(B)** P815 cells were preincubated with indicated antibodies (5 µg/ml) for 30 min before coculture with CIK cells. After 5-h coincubation at an E/T ratio of 5:1 in the presence of anti-CD107a-APC and GolgiStop, the percentage of CD107a in bulk CIK cells was determined by FACS. **(C)** P815 cells were preincubated with indicated antibodies (5 µg/ml) for 30 min before coculture with CIK cells. After 20-h coincubation at an E/T ratio of 20:1, IFN-γ level in supernatant was determined by sandwich ELISA. **(D)** Conjugate assay was performed as described in against antibody-loaded P815 cells at an E/T ratio of 5:1. The data are represented as mean ± SD of triplicates per condition and one representative of at least three independent experiments. *p < 0.05, ****p < 0.0001, ns, not significant, calculated by one-way or two-way ANOVA, Bonferroni’s *post-hoc* test.

To further investigate the possible association of NKG2D and 2B4 in CIK cells, we used the mouse mastocytoma cell line (P815), which expresses the Fc receptor on the surface that can easily recognize and bind the respective antibodies, e.g., anti-NKG2D, anti-2B4, in our experimental setup. Compared to the IgG1 treatment (**Figure 3A**), lysis was significantly increased when anti-NKG2D was preloaded on P815 cells, while it remained unchanged as anti-2B4 was loaded. Of note, no additional improvement in lysis occurred when NKG2D and 2B4 were combined in contrast to NKG2D ligation alone. Moreover, anti-CD3, which showed high sensitivity and lysis rate as a positive control, did not further increase lysis when being co-incubated (CD3 and NKG2D; CD3 and 2B4). NKG2D ligation could increase degranulation, IFN-γ secretion, and E/T conjugate formation, whereas 2B4 alone or in combination with NKG2D had minor to no effect (**Figures 3B–D**). Hence, we confirm that NKG2D is an activating receptor on CIK cells that can promote cytotoxicity, E/T conjugate formation, IFN-γ production, and degranulation. In contrast, 2B4 ligation appears to weakly induce cytotoxicity of CIK cells and does not contribute to E/T conjugation, IFN-γ secretion, and degranulation. Additionally, 2B4 provided no synergy in these CIK cell-mediated activities with NKG2D.

### NKG2D Contributes Alone to Degranulation, IFN-γ Secretion, and LFA-1 Activation, Whereas 2B4 Only Provides Synergistic Effect in Activation of LFA-1

It is worth mentioning that even in the absence of any antibodies, CIK cells were still able to form a tight conjugation with P815 cells and induce cytotoxicity (**Figures 3A, D**), suggesting that P815 cells themselves can be recognized and killed by CIK cells. Therefore, the impact of these yet to be known P815-CIK intrinsic interactions is difficult to exclude from our data. To avoid this potential interference, we used monoclonal antibodies against these receptors (NKG2D and 2B4) by a plate-bound method or by cross-linking with a rabbit anti-mouse antibody to define the relative contribution of NKG2D and 2B4 in degranulation, IFN-γ secretion, and LFA-1 activation.

**Figure 4:**
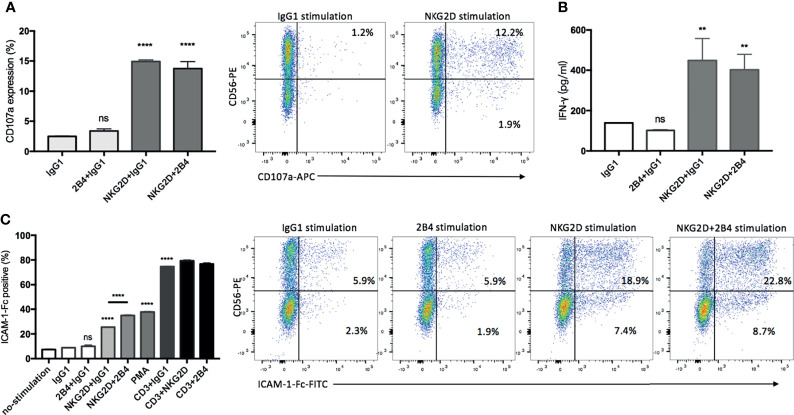
NKG2D contributes alone to degranulation, IFN-γ secretion, and LFA-1 activation, whereas 2B4 provides synergistic signals. **(A)** CIK cells were stimulated by indicated plate-bound antibodies (5 µg/ml) for 5 h in the presence of anti-CD107a-APC and GolgiStop, and the percentage of CD107a on CIK cells was determined by staining cells with anti-CD3-FITC and anti-CD56-PE. FACS dot plots showing the representative degranulation of different CIK subsets (within CD3+ gate) in response to IgG1 or NKG2D stimulation. **(B)** After 20 h of stimulation by indicated plate-bound antibodies (5 µg/ml), IFN-γ was quantified using supernatant by sandwich ELISA. **(C)** CIK cells were unstimulated or stimulated with PMA or indicated antibodies in pairwise combination or combined with IgG1 isotype control. Following the cross-link of the receptors with goat F(ab)2 anti-mouse IgG, the activation of LFA-1 was measured by staining with ICAM-1–Fc complexes. For gating out CIK subsets, cells were stained with anti-CD3-APC and anti-CD56-PE after ICAM-1–Fc complexes staining. Dot plots showing the representative LFA-1 activation on different CIK subsets (within CD3+ gate) in response to indicated stimulations. The data are represented as mean ± SD of triplicates per condition and one representative of three independent experiments. **p < 0.01, ****p < 0.0001, ns, not significant calculated by one-way ANOVA, Bonferroni’s *post-hoc* test.

The analysis revealed that NKG2D alone was effective enough to induce degranulation and IFN-γ secretion, while 2B4 alone lacked this ability and led to no further improvement in combination with NKG2D (**Figures 4A, B**). As expected, the majority of degranulated cells that responded to anti-NKG2D were found to be the CD3+CD56+ subpopulation (**Figure 4A**, dot plots). To further verify our results, PBMCs were treated under the same condition as a positive control, since NK cells have been reported to respond well to costimulatioin of NKG2D and 2B4 (32). After stimulation, NK cells were identified by staining with CD3 and CD56 surface markers. As expected, the individual engagement of NKG2D or 2B4 led to minor degranulation of NK cells, whereas the coengagement of both receptors resulted in a strong synergy (**Figure S2**).

Considering the activation of LFA-1 being influenced by the inside-out signals from other activating receptors in NK cells (31, 33), we performed a ligand complex-based adhesion assay (LC-AA) to examine the effect of NKG2D and 2B4 individually and in combination on the activation of LFA-1 in mature CIK cells. Consistent with others (31), LC-AA assay is able to detect the conformational changes in both avidity and affinity of LFA-1 on CIK cells, as the m24 clone antibody intensively detected the high-affinity conformation of LFA-1 induced by Mg^2+^ with minimal staining in cells stimulated by PMA, which has been reported to induce the avidity rather than the affinity of LAF-1, while LC-AA detected the activation of LFA-1 induced either by Mg^2+^ or PMA (**Figure S3A**). By using LC-AA assay, we found that in the absence of any stimulation (e.g., by IgG1), only a few LFA-1 molecules were active on the surface of CIK cells (**Figure 4C**). NKG2D alone was able to induce LFA-1 activation, and some synergy occurred when it was combined with 2B4, but 2B4 ligation solely failed to induce any activation. Additionally, NKG2D or 2B4 in combination with anti-CD3 did not significantly improve LFA-1 activation. Of interest, after staining with CD3 and CD56 markers, we observed that both CD3+CD56+ and CD3+CD56− subsets were responsible to the stimulation of NKG2D in LFA-1 activation, while CD3+CD56+ cells showed a stronger response than the counterpart CD3+CD56− cells (**Figure 4C**, dot plots). Moreover, the effect of costimulation of NKG2D and 2B4 on LFA-1 activation appeared to be primarily attributed to the CD3+CD56+ subset. To mention, as shown previously (31), we also observed contrasting results with resting NK cells, i.e., 2B4 ligation resulted in a stronger activation of LFA-1 compared to NKG2D ligation (**Figure S3B**).

Hence, NKG2D alone is sufficient to induce not only degranulation and IFN-γ secretion but also activation of LFA-1 in CIK cells, while 2B4 provides only synergistic signal in activation of LFA-1.

### CD3+CD56+ Subset Is the Predominant Population in CIK Cells in Response to NKG2D Ligation

**Figure 5:**
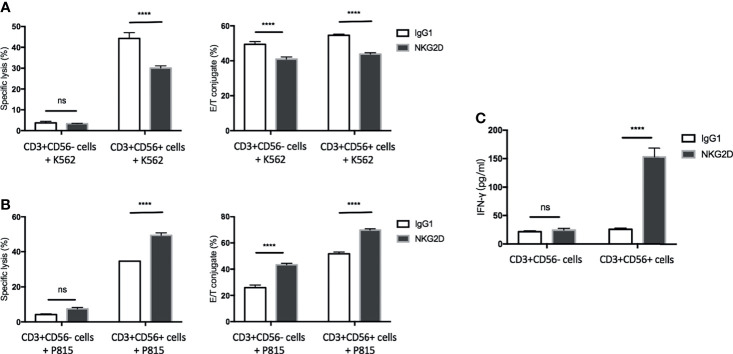
The CD3+CD56+ subset is the predominant population in CIK cells in response to NKG2D ligation. CIK cells were sorted by MACS separation using CD56-conjugated microbeads. **(A)** Blocking cytotoxicity (20:1 E/T ratio) and conjugate (5:1 E/T ratio) assays were performed as described in by using sorted CD3+CD56− cells or CD3+CD56+ cells against K562 cells. **(B)** Redirected cytotoxicity (20:1 E/T ratio) and conjugate (5:1 E/T ratio) assays were performed as described in by using sorted CD3+CD56− cells or CD3+CD56+ cells against antibody-loaded P815 cells (5 µg/ml). **(C)** After 20 h of stimulation by indicated plate-bound antibodies (5 µg/ml), IFN-γ produced by CD3+CD56− cells or CD3+CD56+ cells was quantified using supernatant by sandwich ELISA. The data are represented as mean ± SD of triplicates per condition and one representative of three independent experiments. ****p < 0.0001, ns, not significant calculated by two-way ANOVA, Bonferroni’s *post-hoc* test.

CD3+CD56+ cells have been considered the population dominating the CIK cell-mediated activities (3). In line with this notion, the previous section of our study has shown that the CD3+CD56+ subset is the major population for degranulation and activation of LFA-1 in response to NKG2D stimulation. To further demonstrate its predominant contribution upon NKG2D ligation, mature CIK cells were sorted by MACS separation using CD56-conjugated microbeads. Subsequently, the cytotoxicity, conjugate formation and IFN-γ secretion assays were performed by using sorted CD3+CD56+ and CD3+CD56− cells against K562 or P815 cell line or stimulated by plate-coated antibodies. As expected, CD3+CD56− cells mediated minimal cytotoxicity against K562 or P815 cells led to minor to no change in lysis upon either blocking or redirecting NKG2D receptor, while CD3+CD56+ cells displayed robust cytotoxic capacity against both cell lines (**Figures 5A, B**, left bar graph, respectively). In agreement with the results from experiments using bulk CIK cells, the cytotoxicity of CD3+CD56+ subset was significantly attenuated against K562 cells after blocking NKG2D and was dramatically increased against NKG2D-redirected P815 cells. In addition, similar finding was observed in IFN-γ secretion (**Figure 5C**), showing that only the CD3+CD56+ subset responded to the NKG2D stimulation and led to IFN-γ secretion. Intriguingly, the influence of NKG2D engagement on E/T conjugation was observed in both subsets. Blockade of NKG2D on both subpopulations led to a decrease in conjugation formation against K562 cells (**Figure 5A**, right bar graph). Conversely, an augment in E/T contact was observed in both subsets when cocultured with NKG2D-redirected P815 cells (**Figure 5B**, right bar graph). These results are consistent with those from **Figure 4C** showing that NKG2D stimulation led to LFA-1 activation on both CD3+CD56+ and CD3+CD56− cells. Overall, we show that the CD3+CD56+ subset is the predominant contributor in CIK cells responsible for the NKG2D-mediated cytotoxicity, degranulation, and IFN-γ secretion. In contrast, NKG2D ligation failed to induce these activities but was able to promote the E/T conjugate formation and LFA-1 activation in the CD3+CD56− subset.

### PI3K, PLC-γ, and Src Are Involved in the NKG2D-Mediated Activities in CIK Cells

**Figure 6:**
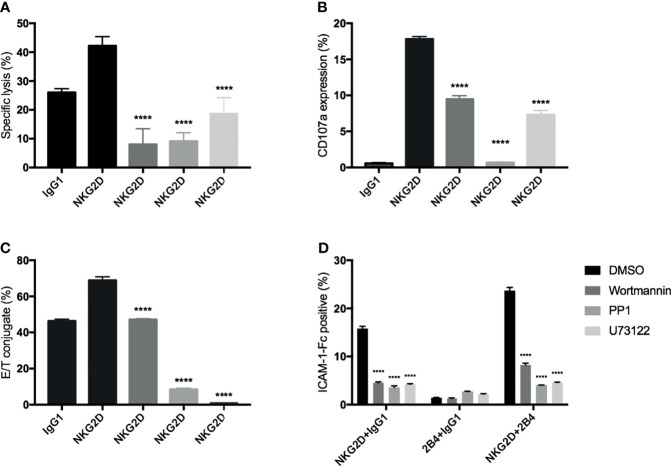
Signaling inhibition by chemical inhibitors to block the NKG2D-mediated activities in CIK cells. Bulk CIK cells were preincubated with various signaling inhibitors (DMSO control, wortmannin [PI3K inhibitor], PP1 [Src kinase inhibitor], and U73122 [phospholipase C-γ inhibitor]) and then stimulated with P815 cells preloaded with indicated antibodies (5 µg/ml) **(A–C)** or stimulated with anti-NKG2D, anti-2B4, or both (1 µg/ml) by cross-linking the receptors with goat F(ab)2 anti-mouse IgG **(D)**. **(A)** Cytotoxicity assay was performed as described in **Figure 3A**. **(B)** Degranulation was performed as described in **Figure 3B**. **(C)** Conjugate assay was performed as described in **Figure 3D**. **(D)** The activation level of LFA-1 was measured by staining with ICAM-1–Fc complexes. The data are represented as mean ± SD of triplicates per condition and one representative of three independent experiments. ****p < 0.0001 calculated by one-way or two-way ANOVA, Bonferroni’s *post-hoc* test.

In NK cells, PI3K-, PLC-γ-, and Src-related signaling pathways have been reported to be associated with NKG2D (or 2B4)-mediated activation (31, 34, 35). Therefore, to enhance our findings, we used three inhibitors: wortmannin (PI3K pathway), PP1 (Src pathway), and U73122 (PLC-γ pathway) to interfere with these known signaling pathways in our experimental settings. We found that all these inhibitors significantly inhibited the cytotoxicity, degranulation, and E/NKG2D-redirected P815 cells (**Figures 6A–C**) and strongly blocked the induced by NKG2D alone and in combination with 2B4 (**Figure 6D**). These suggest that, similar to NK cells, these specific signaling pathways (PI3K, PLC-γ, and Src) may also play a role in NKG2D-induced activities in CIK cells.

## Discussion

In the current study, we investigated the role of NKG2D and 2B4 individually or in combination in CIK cell-related activities. Consistent with previous studies (14, 15), we showed that the percentage of CD3+CD56+ CIK cells and the expression of NKG2D were significantly increased during the 14-day culture. Of note, as the CIK cells matured, they exhibited higher 2B4 expression. One previous study failed to detect 2B4 on CIK cells (36); however, the use of different clones for the 2B4 marker could be a possible reason for this discrepancy. To ensure consistency in our current study, we stained the cells with a common clone (C1.7) against 2B4, as it is used in most NK-associated studies (32, 35, 37). As expected, we observed that all NK cells expressed 2B4 when we analyzed the phenotype of freshly isolated PBMCs at day 0, thus confirming its expression on CIK cells.

As expected, the blockade of NKG2D partially attenuated the cytolysis and E/T conjugate against K562 cells. Additionally, redirecting P815 cells against NKG2D led to a significant increase in CIK cell-mediated cytotoxicity, E/T conjugate formation, and degranulation. In agreement with previous studies (15, 20), these data support the idea that NKG2D acts as an activating receptor for CIK cells and also imply that it may provide signals to activate LFA-1 on CIK cells that can enhance the E/T contact.

To further determine whether ligation of NKG2D alone is sufficient to activate CIK cells, we used monoclonal antibodies against NKG2D by the plate-bound method or by cross-linking with a secondary antibody. We demonstrated that NKG2D engagement was sufficient to induce degranulation and IFN-γ secretion from CIK cells on its own. The cytolytic activity of CIK cells is dependent on the cell-to-cell contact, as Mehta et al. (14) demonstrated that the killing could be completely blocked by anti-LFA-1 antibody. However, we found that active LFA-1 was virtually undetectable on CIK cells during the expansion. Normally, LFA-1 has varying avidities and affinities for its ligand ICAMs, depending on the conformation and clustering of LFA-1, and modifications in them have been shown to be influenced by the inside-out signals from other activating receptors in T and NK cells (31, 33, 38). Consistent with this, here we showed that LFA-1 could also be activated by the engagement of other molecules on CIK cells. More specifically, NKG2D engagement alone could induce the LAF-1 activation on mature CIK cells through similar signaling pathways (PI3K, PLC-γ, and Src) to NK cells (31, 34, 35). Of interest, it has been reported that the resting NK cells barely responded to single NKG2D stimulation, whereas IL-2-activated NK cells responded well (24, 32). Therefore, we speculate that the responsiveness of CIK cells to the NKG2D engagement might be attributed to the presence of IL-2 in CIK culture, as it was previously shown that only cells cultured in high-dose IL-2 expressed DAP10 and were cytotoxic (15) and the incubation with cytokine could make effector cells more susceptible to the subsequent activating receptor stimulation (31). Taken together, we highlight that NKG2D alone is adequate enough to activate CIK cells, inducing degranulation, IFN-γ secretion, and LFA-1 activation.

It is worth mentioning that although ligation of other receptors can induce activation of LFA-1, making it more active toward its ICAM ligands, still a higher rate of E/T conjugation may not always reflect a better immune response. To some extent, this can be seen in **Figure 2D**, where the NK-resistant Raji cells formed a significantly higher E/T binding rate compared to the NK-sensitive K562 cells. On a broader aspect, this may be explained by the dynamic regulatory process of E/T conjugation (involving both attachment and detachment systems), where an activating signal not only promotes E/T contact but also accelerates dissociation (39).

Strikingly, the scenario appears to be different in 2B4 compared to NKG2D, with no effect of 2B4 blockade on cytotoxicity and E/T conjugate against SU-DHL-4 cells, and no increase in the redirected cytotoxicity, degranulation, and E/T binding against 2B4-loaded P815 cells. Similarly, in the experiments using the plate-bound stimulation or by cross-linking receptors with a secondary antibody, 2B4 alone was not sufficient enough to modulate activities of CIK cells and hence was incapable of inducing the degranulation, IFN-γ secretion, and LFA-1 activation. Here, we speculate that a chronic exposure to neighboring CD48+ CIK cells (during *ex vivo* expansion) might make 2B4+ CIK cells more fatigued to react on secondary stimulation. One early study (40) reported that the 2B4-mediated signal in NK cells was defined solely by the tyrosine-based motifs in the cytoplasmic domain of 2B4, and it was not appreciably influenced by the transmembrane and extracellular segments of 2B4. This may also occur in CIK cells and further studies are needed. In most cases of our study, 2B4 stimulation failed to produce synergistic signals to NKG2D, except in the activation of LFA-1, suggesting that the synergy of NKG2D and 2B4 indeed exists in CIK cells but it is confined to certain functional responses (e.g., LFA-1 activation).

In the current study, we also demonstrated that most activities (including cytotoxicity, degranulation, IFN-γ secretion, and LFA-1 activation) upon NKG2D engagement were conducted by CD3+CD56+ subset, which is in agreement with a previous study showing that CD3+CD56+ cells are the main MHC-unrestricted cytotoxic population of bulk CIK cells (3). In contrast, NKG2D ligation was unable to activate the counterpart CD3+CD56− subset and failed to induce the cytotoxicity, degranulation, and IFN-γ release, whereas it induced LFA-1 activation on this population and made an impact on E/T conjugate formation. Our finding is consistent with previous reports, which have shown that ligation of NKG2D was able to induce T cells to form an immune synapse with target cells in the absence of TCR stimulation (41, 42). As expected, similar to NK cells, signaling pathways (PI3K, PLC-γ, and Src) appear to be involved in NKG2D-induced activities in CIK cells, as the relevant inhibitors strongly inhibited the cytotoxicity, degranulation, E/T conjugate, and LFA-1 activation.

Contrary to previous findings that NKG2D provides strong synergistic effects with 2B4 in NK cells and costimulatory signals with the TCR–CD3 complex in CD8 cytotoxic T cells (25, 31, 32), in our current study, we report that NKG2D provides limited synergy with 2B4 but no significant costimulation with CD3 in activating CIK cells. There were similar results presented by Verneris MR et al. (15), which also showed that NKG2D did not produce synergy with CD3 in P815 redirected cytotoxicity. Despite the fact that CIK cells share phenotypic and functional characteristics with NK and T cells, a divergence in receptor function can be assumed. One early study showed that NKG2D barely provided additional activation of iNKT cells when combined with anti-CD3 stimulation (43). Likewise, 2B4 alone failed to induce the degranulation of iNKT cells (43). In this regard, *ex vivo* expanded CIK cells seem to function in a similar manner to the classical iNKT cells with deficiencies in 2B4 stimulation and costimulation of CD3 with NKG2D. A complete understanding of the interaction between these molecules is warranted, as aforementioned, NKG2D produces strong synergy with 2B4 in NK cells and costimulatory signals with the TCR–CD3 complex in T cells, providing the opportunity to enhance the CD3ζ-based chimeric antigen receptor (CAR) construct for NK or T cells by integration of NKG2D or 2B4 (44, 45). Recently, CAR-CIK cells have also been explored with some encouraging results; however, further validation will be required to draw conclusions (46–48). Given the divergence in receptor function between CIK cells and NK (or T) cells as we presented here, the CAR structure for CIK cells may need to be established independently to maximize their functional potential.

Nevertheless, herein, we show that NKG2D ligation alone is able to induce the activation of CIK cell, supporting the idea of targeting the NKG2D axis holds great potential for enhancing the antitumor activity of CIK cells. Given the wide distribution of NKG2D ligands in malignancies, CIK cells appear to be endowed with a broad antitumor spectrum (i.e., in an NKG2D-dependent manner). However, NKG2D ligands have been reported to be lost from the tumor surface either by proteolytic shedding or exosome excretion (49, 50). Therefore, the strategy for preventing surface loss or upregulating the expression of NKG2D ligands may help to enhance the cytolytic ability of CIK cells against these tumors. We have previously shown that the cytotoxicity of CIK cells was enhanced by stabilization of MICA/B on tumor cells through selective small-molecule inhibitors or a specific anti-MICA monoclonal antibody (51, 52). Furthermore, the ligands of NKG2D can be induced or upregulated by chemotherapeutic agents or radiation therapy (53, 54); thus, the combination therapy of CIK cells with these traditional modalities might have synergistic benefits for cancer patients and further studies are needed in this regard.

In conclusion, herein we demonstrate that the engagement of NKG2D alone is sufficient to activate CIK cells without additional costimulatory signals, thereby strengthening the idea of targeting the NKG2D axis might be a promising approach to improve CIK cell therapy. In addition, CIK cells appear to function similarly to the classical iNKT cells with deficiencies in 2B4 stimulation and in costimulation of CD3 with NKG2D, but exhibit divergence in receptor function compared to NK and T cells.

## Data Availability Statement

The raw data supporting the conclusions of this article will be made available by the authors, without undue reservation.

## Ethics Statement

Ethical review and approval were not required for the study on human participants in accordance with the local legislation and institutional requirements. Written informed consent for participation was not required for this study in accordance with the national legislation and the institutional requirements.

## Author Contributions

Conceptualization: XW and IS-W. Methodology: XW. Software: IS-W. Validation: XW and IS-W. Formal analysis: XW, AS, JO, and IS-W. Data curation: XW, AS, JO, and IS-W. Writing—original draft preparation: XW. Writing—review and editing: all co-authors. Visualization: XW and AS. Supervision: IS-W, HW, ME, and DS. Project administration: IS-W. All authors contributed to the article and approved the submitted version.

## Conflict of Interest

The authors declare that the research was conducted in the absence of any commercial or financial relationships that could be construed as a potential conflict of interest.

## Publisher’s Note

All claims expressed in this article are solely those of the authors and do not necessarily represent those of their affiliated organizations, or those of the publisher, the editors and the reviewers. Any product that may be evaluated in this article, or claim that may be made by its manufacturer, is not guaranteed or endorsed by the publisher.

## Supplementary Material

Figure S1 NKG2D and 2B4 expression on NK cells. NKG2D and 2B4 expression on freshly isolated NK cells from PBMCs were measured by flow cytometry after staining with anti-CD3-PerCP-Cy5.5, anti-CD56-FITC, anti-NKG2D-APC and anti-2B4-PE antibodies. Dot plots show the phenotype of freshly isolated lymphocytes (left) and the expression of NKG2D (79.2%) and 2B4 (99.6%) within CD3-CD56+ NK cell population (right).

Figure S2 Costimulation of NKG2D and 2B4 induces strong synergy in degranulation of NK cells. PBMCs were stimulated by indicated plate-bound antibodies (5 µg/ml) for 5 h in the presence of anti-CD107a-APC and GolgiStop. At the end of culture, cells were stained with anti-CD3-FITC and anti-CD56-PE for selection of NK population. The percentage of CD107a on NK cells was determined by FACS. Dot plots showing the percentage of CD3-CD56+ NK population in PBMCs (far left) and the percentage of CD107a expression in NK cells from two donors.

Figure S3 LC-AA is compared with anti-LFA-1 antibody (clone, m24) staining in CIK cells and detects the LFA-1 activation on NK cells. **(A)** Incubation of CIK cells with a high Mg^2+^ ion concentration induces high affinity of LFA-1, resulting in a strong staining in both assays. Treatment with PMA has been reported to induce high avidity of LFA-1 without affecting the affinity. Cells treated with PMA show an intermediate binding of the ICAM-1-Fc complexes in the LC-AA, but hardly any staining with the m24. One representative of 3 independent experiments is shown. **(B)** Freshly isolated PBMCs cells were stimulated with indicated antibodies in pairwise combination or combined with IgG1 isotype control. Following the crosslink of the receptors with goat F(ab)2 anti-mouse IgG, the activation of LFA-1 was measured by staining with ICAM-1-Fc complexes. For gating out CD3-CD56+ NK population, cells were stained with anti-CD3-APC and anti-CD56-PE after ICAM-1-Fc complexes staining. The data are represented as mean ± SD of triplicates per condition and one representative of three independent experiments. ****p < 0.0001 calculated by one-way ANOVA, Bonferroni’s *post-hoc* test.
