# Supplementary figures and images for: NKG2D Engagement Alone Is Sufficient to Activate Cytokine-Induced Killer Cells While 2B4 Only Provides Limited Coactivation
Source: Front Immunol. 2021 Oct 7;12:731767. doi: 10.3389/fimmu.2021.731767 (PMC8529192; doi:10.3389/fimmu.2021.731767)

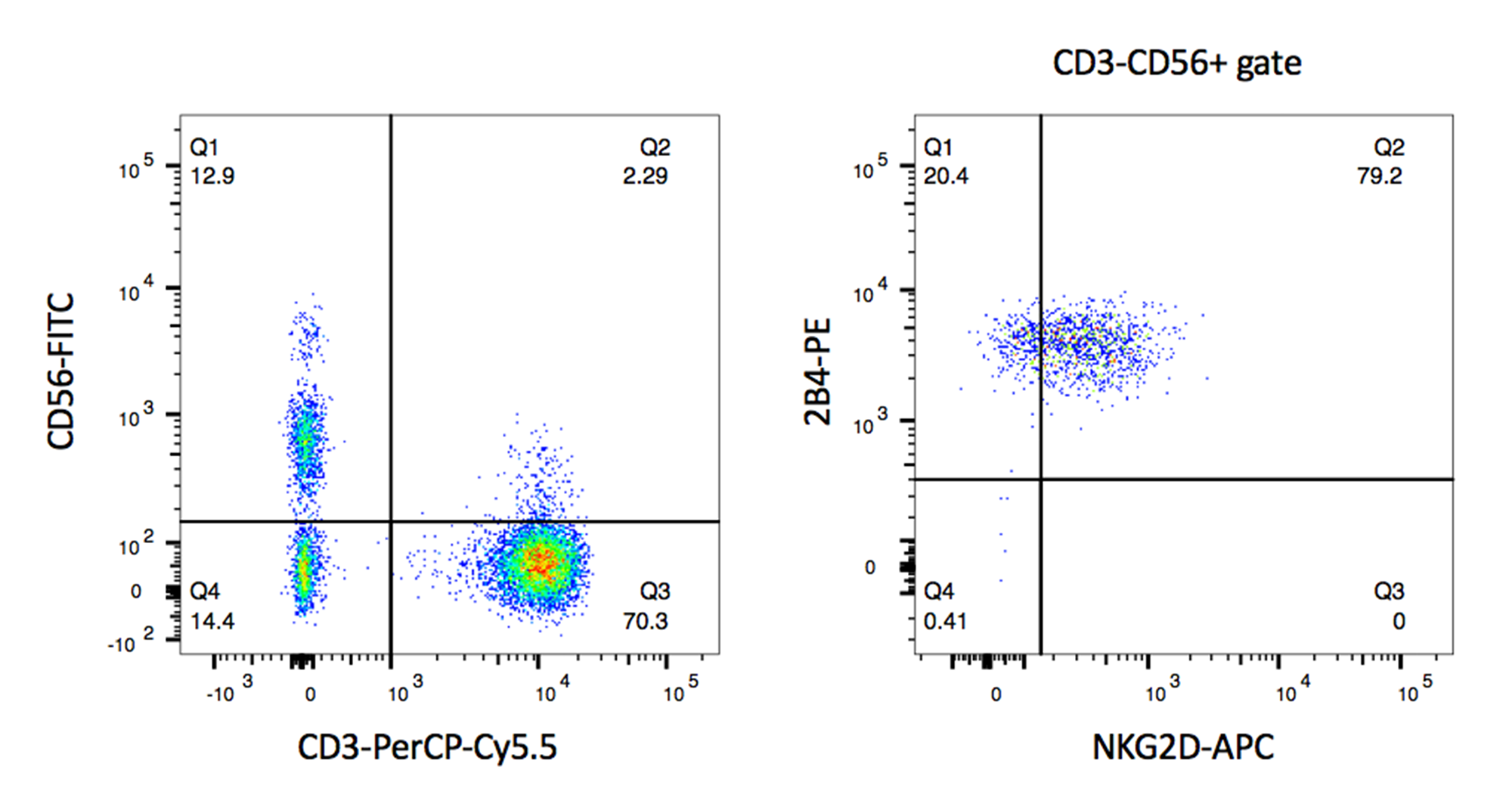

Supplement: Figure S1 — NKG2D and 2B4 expression on NK cells. NKG2D and 2B4 expression on freshly isolated NK cells from PBMCs were measured by flow cytometry after staining with anti-CD3-PerCP-Cy5.5, anti-CD56-FITC, anti-NKG2D-APC and anti-2B4-PE antibodies. Dot plots show the phenotype of freshly isolated lymphocytes (left) and the expression of NKG2D (79.2%) and 2B4 (99.6%) within CD3-CD56+ NK cell population (right). [file Image_1.tif]

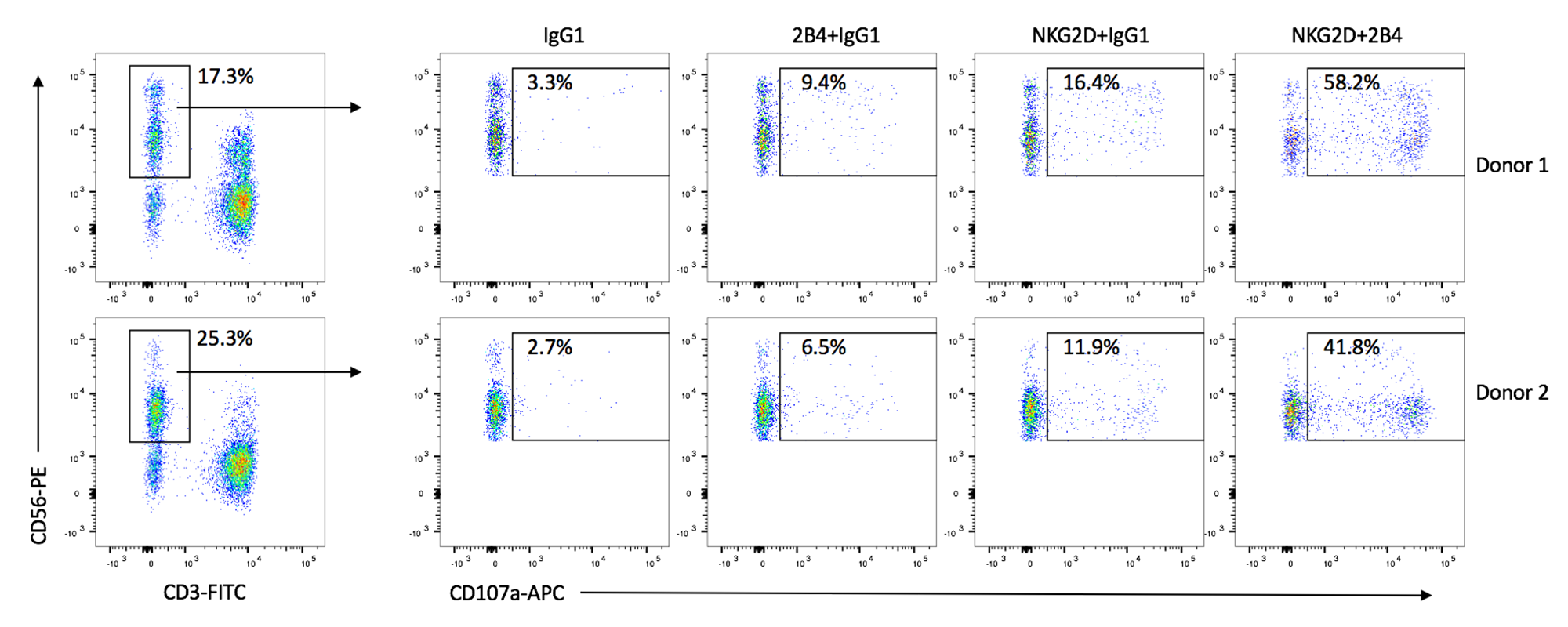

Supplement: Figure S2 — Costimulation of NKG2D and 2B4 induces strong synergy in degranulation of NK cells. PBMCs were stimulated by indicated plate-bound antibodies (5 µg/ml) for 5 h in the presence of anti-CD107a-APC and GolgiStop. At the end of culture, cells were stained with anti-CD3-FITC and anti-CD56-PE for selection of NK population. The percentage of CD107a on NK cells was determined by FACS. Dot plots showing the percentage of CD3-CD56+ NK population in PBMCs (far left) and the percentage of CD107a expression in NK cells from two donors. [file Image_2.tif]

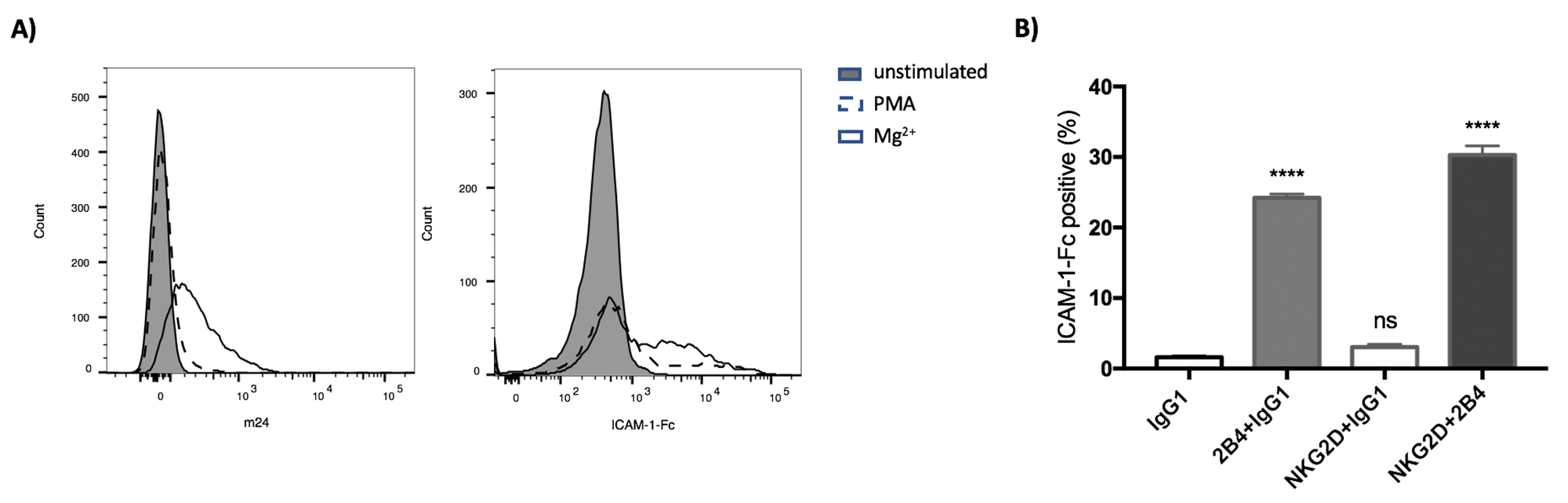

Supplement: Figure S3 — LC-AA is compared with anti-LFA-1 antibody (clone, m24) staining in CIK cells and detects the LFA-1 activation on NK cells. (A) Incubation of CIK cells with a high Mg2+ ion concentration induces high affinity of LFA-1, resulting in a strong staining in both assays. Treatment with PMA has been reported to induce high avidity of LFA-1 without affecting the affinity. Cells treated with PMA show an intermediate binding of the ICAM-1-Fc complexes in the LC-AA, but hardly any staining with the m24. One representative of 3 independent experiments is shown. (B) Freshly isolated PBMCs cells were stimulated with indicated antibodies in pairwise combination or combined with IgG1 isotype control. Following the crosslink of the receptors with goat F(ab)2 anti-mouse IgG, the activation of LFA-1 was measured by staining with ICAM-1-Fc complexes. For gating out CD3-CD56+ NK population, cells were stained with anti-CD3-APC and anti-CD56-PE after ICAM-1-Fc complexes staining. The data are represented as mean ± SD of triplicates per condition and one representative of three independent experiments. ****p < 0.0001 calculated by one-way ANOVA, Bonferroni’s post-hoc test. [file Image_3.tif]
